# Machine Learning in the Design and Performance Prediction of Organic Framework Membranes: Methodologies, Applications, and Industrial Prospects

**DOI:** 10.3390/membranes15060178

**Published:** 2025-06-11

**Authors:** Tong Wu, Jiawei Zhang, Qinghao Yan, Jingxiang Wang, Hao Yang

**Affiliations:** 1 State Key Laboratory of Pollution Control and Resource Reuse, School of the Environment, Nanjing University, Nanjing 210023, China; 522024750027@smail.nju.edu.cn (T.W.); 522024750031@smail.nju.edu.cn (J.Z.); qinghaoyan@smail.nju.edu.cn (Q.Y.); 18662306305@163.com (J.W.); 2 Institute for the Environment and Health, Nanjing University Suzhou Campus, Suzhou 215163, China

**Keywords:** machine learning, organic framework membranes, gas separation, liquid separation, metal–organic framework membranes, covalent organic framework membranes

## Abstract

Organic framework membranes (OFMs) have emerged as transformative materials for separation technologies due to their tunable porosity, structural diversity, and stability, yet their design and optimization face challenges in navigating vast chemical spaces and complex performance trade-offs. This review highlights the pivotal role of machine learning (ML) in overcoming these limitations by integrating multi-source data, constructing quantitative structure–property relationships, and enabling the cross-scale optimization of OFMs. Methodologically, ML workflows—spanning data construction, feature engineering, and model optimization—accelerate candidate screening, inverse design, and mechanistic interpretation, as demonstrated in gas separations and nascent liquid-phase applications. Key findings reveal that ML identifies critical structural descriptors and environmental parameters, guiding the development of high-performance membranes that surpass traditional selectivity–permeability limits. Challenges persist in liquid separations due to dynamic operational complexities and data scarcity, while emerging frameworks offer untapped potential. The integration of interpretable ML, in situ characterization, and industrial scalability strategies is essential to transition OFMs from laboratory innovations to sustainable, adaptive separation systems. This review underscores ML’s transformative capacity to bridge computational insights with experimental validation, fostering next-generation membranes for carbon neutrality, water security, and energy-efficient industrial processes.

## 1. Introduction

In recent years, organic framework membranes (OFMs) have garnered significant attention in the field of membrane separation due to their unique structural designability and performance advantages. These materials, constructed through covalent bonds, metal–organic coordination interactions, or intermolecular forces to form porous frameworks, exhibit high porosity, precisely tunable pore sizes, and tailorable microenvironments within their channels [1,2]. Such characteristics enable multiscale design flexibility to address diverse separation requirements, including molecular sieving, ion-selective rejection, and organic compound separation [3,4]. Representative OFMs include metal–organic framework (MOF) membranes [2,5,6], covalent organic framework (COF) membranes [2,7,8], conjugated microporous polymer (CMP) membranes [9], porous organic cage (POC) membranes [10], and porous aromatic framework (PAF) membranes [11,12]. Their core advantages stem from the chemical and topological diversity of building blocks: the selection and assembly of organic linkers allow for the precise regulation of pore size distributions at sub-nanometer to nanometer scales, while pore-wall functionalization optimizes interactions between substances and membrane interfaces. This synergy enhances both separation selectivity and permeation flux, overcoming the traditional “selectivity-permeability trade-off” inherent in conventional membrane materials [13]. Furthermore, the exceptional chemical and thermal stability of OFMs ensures robustness under extreme conditions such as high temperatures, pressures, and aggressive chemical environments (e.g., strong acids/bases), guaranteeing long-term operational reliability [14]. These attributes underscore their broad potential in gas separation, water purification, and energy storage applications.

However, the design and optimization of OFMs face substantial challenges. Despite their theoretically near-infinite chemical space (e.g., over 100,000 experimentally synthesized MOFs [15]), traditional trial-and-error approaches and high-throughput computational screening (HTCS) suffer from high resource consumption and prolonged cycles when evaluating vast candidate libraries. While molecular simulations can predict performance metrics like permeability and selectivity, their computational complexity escalates exponentially with system size, hindering large-scale material exploration. This bottleneck highlights the critical need for data-driven approaches. With the emergence of the “fourth paradigm” of science—Data-Intensive Scientific Discovery [16]—machine learning (ML) has become a pivotal tool for accelerating OFM development [17,18]. ML excels at extracting hidden patterns from existing datasets to establish quantitative structure–property relationships (QSPRs), linking structural descriptors (e.g., pore geometry, functional group distribution) to separation performance [18,19]. This capability enables efficient candidate screening, inverse design guidance, and the seamless integration of simulation with experimental validation. For instance, ML models integrating experimental data, molecular simulations, and theoretical databases can construct multidimensional feature systems, unveil intricate structure–performance correlations, and achieve cross-domain optimization across scenarios such as gas separation [20] and water treatment.

Despite remarkable progress in ML-guided OFM design, current research remains fragmented. On the one hand, the adaptability of ML methodologies to different separation systems (e.g., gas vs. liquid) has yet to be systematically summarized. On the other hand, universal strategies for cross-scale design (from molecular building blocks to macroscopic performance) and industrial translation are still lacking. Additionally, liquid-phase separations, which involve complex mechanisms such as multiphase flow dynamics and interfacial interactions, pose higher challenges in data acquisition and model construction, leaving ML applications in this domain largely nascent. To address these gaps, a comprehensive review is urgently needed to consolidate recent advances in ML-driven OFM design, unify methodologies for diverse scenarios, dissect critical challenges (e.g., data standardization, model interpretability), and outline future directions. This review aims to bridge this gap by providing the first holistic overview of ML innovations in OFM development. By focusing on core applications—gas separation, liquid processing, and molecular diffusion prediction—we elucidate the synergistic mechanisms of data construction, feature engineering, and algorithmic optimization, demonstrating how ML transcends traditional trial-and-error limitations to expedite the rational design and industrial deployment of high-performance OFMs. This work not only serves as a methodological guide for researchers but also injects new momentum into advancing green separation technologies.

## 2. Methodologies and Workflows for Machine Learning

**Figure 1:**
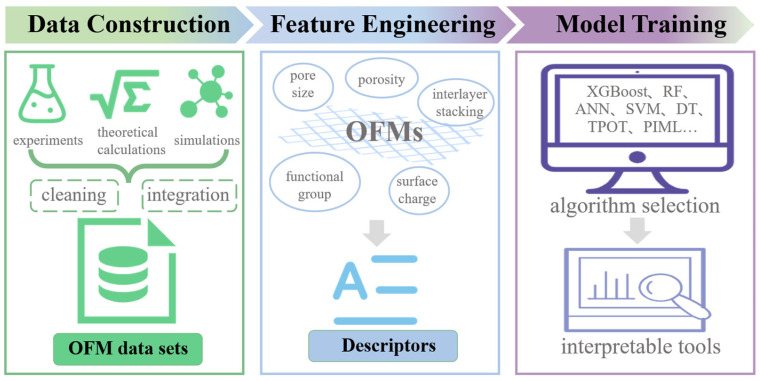
The workflows for machine learning (ML) in organic framework membrane (OFM) design.

The core value of ML in OFM design lies in its ability to systematically integrate multi-source data, resolve complex structural performance relationships, and achieve rational design optimization across scenarios. The process is data-driven and consists of three phases: data construction, feature engineering, and model training with validation (Figure 1).

Data construction is the cornerstone of the approach, and its essence lies in the integration of multidimensional information from experiments, simulations, and theoretical calculations. Experimental data provide actual separation performance parameters and synthesis conditions; molecular simulations generate indicators of microscopic mass transfer behavior; and theoretical databases provide the basis for crystal structure and pore topology analysis. Researchers can search for the required crystal structure files in the database, summarize the macroscopic performance indexes from the literature, and perform calculations using molecular simulations. Performance labels need to be dynamically adapted to different separation needs: gas separation focuses on the balance between selectivity and permeability, water treatment focuses on the synergy between retention and flux, while energy applications require the quantification of ion mobility efficiency and chemical stability. Subsequently, data cleaning is carried out, the core task of which is to repair and normalize the quality of the original data to ensure the consistency and reliability of the data. This includes dealing with missing values, outlier detection and correction, and standardizing data formats and units. The final integration of multi-source data and the verification of the distribution of the cleaned data is reasonable and domain-compliant in order to obtain high-quality, structured OFM data sets to ensure the reliability of the subsequent analysis, and to lay the foundation for the subsequent feature engineering and modeling.

Feature engineering aims to translate the physicochemical properties of OFMs into a machine-readable descriptor system. Geometrical descriptors (pore size distribution, porosity) dominate size sieving effects, chemical descriptors (functional group type, surface charge) regulate substance–membrane interfacial interactions, and topological descriptors (framework connectivity, interlayer stacking) influence mass transfer pathways and mechanical strength [21]. The key parameters are extracted and size differences are eliminated by dimensionality reduction algorithms and feature selection methods, resulting in a high information density input feature set [22]. On this basis, the introduction of environmental parameters (temperature, pressure, and pH) further enhances the dynamic adaptability of the model to actual working conditions.

The model training and validation phase focuses on algorithm selection, performance optimization, and cross-domain migration. The modular ML framework strategically selects algorithms by weighing their strengths and limitations (Table 1): eXtreme Gradient Boosting (XGBoost) achieves top prediction accuracy for large-scale screening but requires careful tuning [23]; Random Forest (RF) offers robust feature interpretability with moderate computational cost [24]; Artificial Neural Networks (ANNs) excel in modeling complex nonlinear relationships, yet demand massive data [25]; Support Vector Machines (SVMs) provide strong generalization for small datasets but scale poorly [26]; Decision Trees (DTs) deliver full transparency yet suffer from instability [24]; while the Tree-based Pipeline Optimization Tool (TPOT) automates pipeline optimization at high computational expense [27]. Beyond conventional data-driven models, Physics-Informed Machine Learning (PIML) embeds domain knowledge, such as governing equations of mass transport or thermodynamic constraints, directly into loss functions [28,29]. This paradigm enhances extrapolation reliability with limited data and ensures physical consistency, showing promise for simulating dynamic membrane processes like fouling evolution or multi-component diffusion. The original input data are first randomly divided into a training set, a validation set, and a test set. The fitted model is trained using the training and validation sets, and finally, the performance of the final model is evaluated on the test set. Hyperparameters are pre-set configuration options for machine learning models (e.g., the decision tree depth, the SVM kernel width, the neural network learning rate) which are not learned directly from data, yet crucially govern model complexity and learning behavior. Tuning them is vital because improper settings cause underfitting or overfitting. Common tuning methods include the following: Grid Search (exhaustively tests predefined combinations), Random Search (samples combinations randomly), and Bayesian Optimization (intelligently selects new parameters based on past evaluations) [30]. For instance, in a study on the accurate prediction of CO_2_ separation performance of metal–organic framework mixed-matrix membranes based on machine learning, researchers conducted hyperparameter tuning of the BP neural network model for robust optimization and validation. By adjusting hyperparameters such as the hidden layer structure, activation function, epochs, and loss function, the predictive performance of the BP neural network model was enhanced [31].

The robust evaluation of model performance relies on rigorous quantitative metrics and validation strategies. For regression tasks predicting membrane properties (e.g., permeability, selectivity, and flux), researchers commonly use the Mean Squared Error (MSE) to quantify the average deviation between predicted and true values (lower is better), and the Coefficient of Determination (R^2^) to indicate how well the model captures data variation (closer to 1 is better) [32]. In classification tasks, precision (the proportion of correctly identified positives, avoiding false alarms) and recall (the proportion of all positives found, avoiding misses) are key evaluation criteria. Model validation relies on cross-validation and interpretable tools (e.g., SHAP analysis [33]) to ensure generalizability and reveal mechanisms for key structural parameters [34]. Crucially, cross-validation involves dividing data into several subsets and iteratively using one for validation, which is essential for assessing model generalization and preventing overfitting to training data.

**Table 1:** Comparison of major machine learning (ML) algorithms.

Algorithm	Advantages	Limitations	Applications	Refs.
eXtreme Gradient Boosting (XGBoost)	Highest accuracy for structured data with efficient computation and built-in feature importance analysis.	Moderate interpretability and sensitivity to noisy data, requiring careful hyperparameter tuning.	High-throughput CO_2_ separation membrane screening; performance optimization.	[23,35,36]
Random Forest (RF)	Strong robustness against overfitting with excellent feature importance interpretability for high-dimensional data.	Computationally expensive for large forests and limited extrapolation capability beyond training ranges.	MOF/COF screening; structure–performance correlation analysis.	[24,37,38]
Artificial Neural Networks (ANNs)	Unmatched flexibility in modeling complex nonlinear relationships and high-dimensional patterns.	Black-box nature reduces interpretability; requires massive data and computational resources to avoid overfitting.	Water permeability/salt rejection modeling; multi-scale separation prediction.	[25,31]
Support Vector Machines (SVMs)	Effective high-dimensional handling with strong generalization via margin maximization and kernel tricks.	Poor scalability to big data and sensitive hyperparameter tuning for nonlinear kernels.	Limited-scale separation performance prediction.	[26,39]
Decision Trees (DTs)	Complete interpretability through intuitive decision rules and low computational cost for small datasets.	Severe overfitting susceptibility and instability with data variations limiting predictive power.	Baseline modeling or constituent learners within RF/XGBoost frameworks.	[24,40]
Tree-based Pipeline Optimization Tool (TPOT)	Automates optimal pipeline selection using genetic algorithms for tree-based methods.	Extremely resource-intensive with limited interpretability of final pipelines.	Efficient identification of top-performing materials from vast chemical spaces.	[27,41,42]

The advantages of this methodological framework are threefold: first, the data-driven alternative to the traditional trial-and-error approach significantly shortens the material development cycle; second, the constructive relationships revealed by the interpretable tools provide theoretical guidance for function-oriented design; and third, the generalized feature system and algorithmic architecture support seamless switching between multiple scenarios. In the future, it is necessary to further integrate in-situ characterization and dynamic working condition data, develop adaptive models, and promote OFMs from laboratory exploration to large-scale applications, so as to inject new impetus into the innovation of green separation technology.

## 3. Machine Learning-Guided Rational Design of OFMs

Against the dual background of global industrialization and energy transition, innovation in separation technology has become a key breakthrough in combating climate change and achieving efficient resource utilization. The multi-component complexity of industrial emission gases, the environmental sensitivity of liquid-phase separations, and the high energy demand for purification of rare gases have imposed demanding performance requirements on membrane materials. Against this background, ML opens up a new dimension for the rational design of OFMs. The optimal structure is selected from tens of thousands of membrane candidates, and the long-term stability under extreme operating conditions is simulated in a virtual environment. This design strategy provides an innovative paradigm for the development of the next generation of high-performance separation membranes.

### 3.1. OFMs for Gas Separations

#### 3.1.1. Carbon Capture

**Figure 2:**
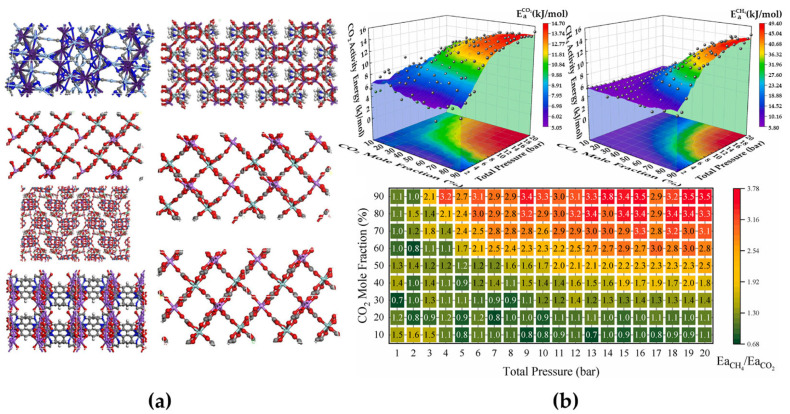
(**a**) The atomistic structures of top-performing metal–organic framework (MOF) membranes: CARGEI, YUJWAD, RIPWEU, VEHNED, WOCJII, YUJWOR, and YUJWUX (the order is from top left, to bottom left, to top right, and finally to bottom right). Reproduced from [35] with Open Access; (**b**) Ea_CH_4__/E_aCO_2__ values exhibit periodic fluctuations rather than smooth variations (e.g., 3.2 at 90% CO_2_ and 4 atm), likely due to hindered CH_4_ diffusion under high CO_2_ concentrations (90%), which amplifies chaotic effects in molecular dynamics (MD) simulations and causes fluctuations in the diffusion coefficient. Reproduced from [25] with Open Access.

Excessive CO_2_ emissions from global fossil fuel consumption have intensified the greenhouse effect, leading to critical ecological challenges such as climate warming and ocean acidification [43]. Carbon capture and storage (CCS), which enables efficient CO_2_ separation from industrial flue gases or direct air capture, is recognized as a critical pathway toward achieving carbon neutrality [44]. OFMs, with their tunable sub-nanometer channels and chemisorption sites, offer unique advantages for the precise sieving of CO_2_/N_2_ and CO_2_/CH_4_ gas mixtures. Nevertheless, optimizing OFMs for complex operational conditions, such as fluctuating flue gas humidity and multicomponent competitive adsorption, remains challenging. Traditional trial-and-error approaches struggle to navigate the vast chemical design space, while the computational costs of molecular simulations limit high-throughput screening scalability. ML addresses these challenges by integrating material structural encoding, separation performance databases, and cross-scale modeling, thereby establishing a data-driven paradigm for designing next-generation CO_2_ separation membranes with enhanced stability and energy efficiency. Recent advancements in pure MOF membranes highlight the potential of ML-guided design. Situ et al. [35] analyzed 6013 experimental MOF membranes through grand canonical Monte Carlo and molecular dynamics (MD) simulations, revealing that gas permeability correlates strongly with structural descriptors such as the available permeation area. By employing XGBoost, they demonstrated the impact of the available permeation area once again and screened seven high-performance MOFs (Figure 2a). Similarly, Zhang et al. [45] developed a filler database of 8167 IL@MOF composites and utilized RF models to demonstrate that the accessible volume and gravimetric surface area dominate CO_2_/N_2_ separation performance. Their experimental validation of [NH_2_-Pmim][Tf_2_N]@ZIF-67 membranes showcases the synergy between computational predictions and empirical verification. Despite the promise of pure MOF membranes, their practical application is often hindered by mechanical fragility and scalability challenges. This has spurred growing interest in mixed-matrix membranes (MMMs), which integrate MOFs into polymer matrices to balance selectivity with processability [46]. Recent studies exemplify the role of ML in accelerating MMM development: Cheng et al. [25] combined molecular simulations with ANNs to optimize CO_2_/CH_4_ separation in IRMOF-1-based membranes, achieving high prediction accuracy (R^2^ = 0.982) (Figure 2b); Guan et al. [37] employed RF to identify MOFs with pore sizes >1 nm and surface areas ~800 m^2^/g, leading to Cu-CAT-1 and Cu-THQ MMMs that exceeded the 2008 Robeson upper bound; Alizamir et al. [32] developed a hybrid extreme learning machine model based on the extreme learning machines algorithm optimized with the BAT optimization algorithm, revealing the MOF cage size and polymer type as critical factors for CO_2_ permeability; Yao et al. [31] utilized genetic algorithm-optimized ANNs to predict the CO_2_ permeability and CO_2_/N_2_ selectivity of MOF MMMs. The study derived from the SHAP algorithm that the MOF type and polymer type are the most important factors for membrane permeability and selectivity, respectively; and Wan et al. [47] screened 54,117 polymer–MOF combinations via ensemble models, emphasizing the pore limit diameter and fractional free volume as key parameters. Collectively, these efforts demonstrate ML’s capacity to decode structure–performance relationships, accelerate material discovery, and bridge computational insights with experimental validation, ultimately advancing industrially viable CO_2_ separation technologies.

#### 3.1.2. Hydrogen Separation

**Figure 3:**
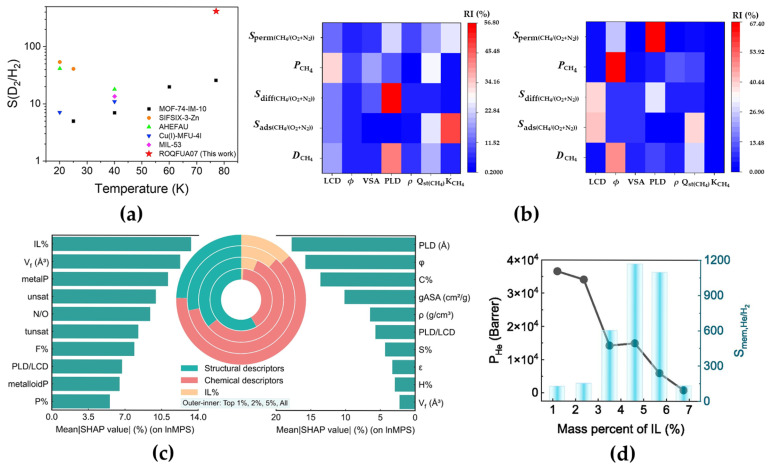
(**a**) A comparative study of D_2_/H_2_ separation selectivity between ROQFUA07 and other MOF materials. Reprinted with copyright permission from Ref. [39] Copyright 2019 Elsevier B.V.; (**b**) relative importance values of the seven descriptors predicted by the RF algorithm for CH_4_/O_2_ + N_2_ (left) and H_2_/O_2_ + N_2_ (right). Reproduced from [40] with Open Access; (**c**) the descriptor importance for lnMPS, comparing the top 1% IL@APMOF set (left bar chart) with the entire IL@APMOF set (right bar chart). The SHAP values are listed on the horizontal axis. The circular diagrams show the distribution and relative importance of structural descriptors (green), chemical descriptors (red), and the ionic liquid content (IL%) (yellow) across various datasets, progressing inward from the outer circle (representing the top 1% IL@APMOFs) to the top 2%, top 5%, and the entire IL@APMOF dataset. Reprinted with copyright permission from Ref. [50] Copyright 2025 Elsevier B.V.; (**d**) the relationship between the IL mass percent and the performance metrics of the IL@APMOF composite membrane, including He permeability and membrane selectivity. Reprinted with copyright permission from Ref. [50] Copyright 2025 Elsevier B.V.

As a clean energy carrier, hydrogen plays a pivotal role in decarbonizing industries and enabling sustainable energy transitions. Efficient hydrogen recovery from gas mixtures (e.g., syngas, natural gas, or industrial byproducts) is critical for maximizing its utilization, yet conventional separation methods often suffer from high energy costs and limited selectivity [48]. OFMs, particularly MOFs, have emerged as promising candidates due to their tunable pore architectures and surface chemistries. Recent advances in ML further empower researchers to navigate the vast design space of MOF membranes, accelerating the discovery of high-performance materials tailored for hydrogen purification and recovery. Recent studies demonstrate ML’s efficacy in optimizing MOF membranes for hydrogen separation from multicomponent gas streams. For instance, Zhou et al. [39] integrated physics-based modeling with ML to screen 12,723 synthesizable MOFs for D_2_/H_2_ separation, identifying the pore limit diameter (PLD) and largest cavity diameter (LCD) as decisive structural features (Figure 3a). Similarly, Bai et al. [49] developed a novel variable, trade-off multiple selectivity and permeability (TMSP), to evaluate H_2_/*X* (*X* = CH_4_, N_2_, CO_2_) separation in computational-ready MOF membranes. Their Gaussian process regression and RF models achieved high predictive accuracy, with the top candidates surpassing Robeson’s upper bounds for polymer membranes. Extending this approach, Li et al. [40] investigated the performance prediction of MOFs as adsorbents or membranes for capturing hydrogen from air through large-scale computational screening and machine learning approaches. By comparing three ML algorithms (RF, DT, and TPOT), the RF model was identified as the most effective in predicting the performance of H_2_/O_2_ + N_2_ systems. Furthermore, this algorithm revealed that the LCD exhibited the highest relative importance for hydrogen adsorbent performance in the H_2_/O_2_ + N_2_ system. Additionally, five optimal MOF membranes were screened. Analysis of the top-performing MOFs further validated that an LCD close to the kinetic diameter of hydrogen is a critical prerequisite for achieving efficient separation from air (Figure 3b). Beyond multicomponent separation, ML also facilitates specialized hydrogen purification tasks, such as removing trace impurities like helium. Zhang et al. [38] combined molecular simulations with RF models to uncover the PLD and framework porosity as dominant factors in He/H_2_ separation, where electronegative pore surfaces enhance selectivity via quantum sieving effects. He et al. [50] further advanced this field by screening 2873 fluorine-rich ionic liquid@APMOF composites, revealing the ionic liquid content (IL%) as the key driver of separation efficiency (Figure 3c). Their CatBoost-guided optimization yielded tunable IL@APMOF membranes with exceptional He/H_2_ performance (Figure 3d), exemplifying ML’s potential in addressing niche yet high-value separation challenges. Together, these studies underscore ML’s transformative role in bridging computational insights with experimental validation, enabling the rapid identification of MOF membranes that balance selectivity, permeability, and scalability. By decoding structure–performance relationships and automating design iterations, ML-driven strategies are poised to unlock next-generation hydrogen recovery technologies with enhanced efficiency and industrial viability.

#### 3.1.3. Natural Gas Purification

**Figure 4:**
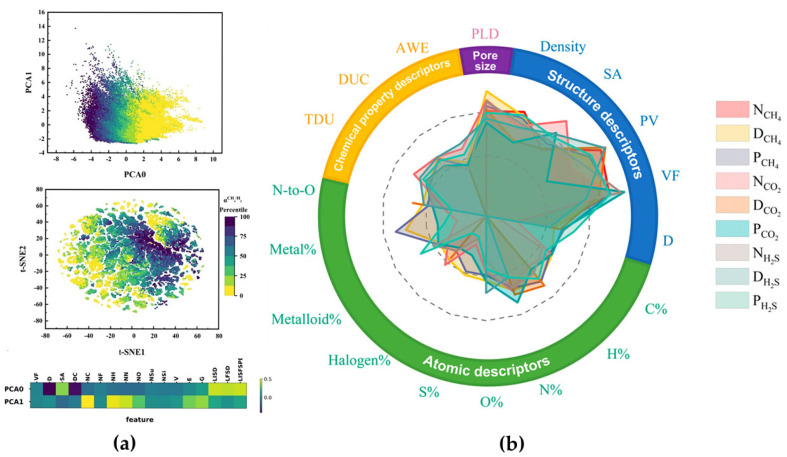
(**a**) Unsupervised learning implemented for classification of adsorption selectivity. Classification results from principal component analysis (PCA) algorithm (top), with color mapping representing the adsorption selectivity percentage and the horizontal and vertical axes representing two principal components, and the T-SNE algorithm (bottom). Weighting map of the descriptors corresponding to the two principal components in the PCA, with color mapping from darker to lighter indicating stronger correlation. Reprinted with copyright permission from Ref. [52] Copyright 2024 American Institute of Chemical Engineers; (**b**) radar plots of feature importance for the gas adsorption, diffusion, and permeability of covalent organic frameworks (COFs). The SHAP values for each type of explainer have been normalized, and the scale range shows a Log distribution from 0.01 to 1. Reprinted with copyright permission from Ref. [41] Copyright 2024 American Chemical Society.

In the field of hydrocarbon separation and natural gas purification, researchers have revealed the deep correlation between the structure and performance of COFs through machine learning. Gulbalkan et al. [51] highlighted recent advances in combining high-throughput molecular simulations and machine learning to accurately identify the most promising MOF and COF membranes among thousands of candidates for the separation of methane from other gases (acetylene, carbon dioxide, helium, hydrogen, and nitrogen). Similarly, Qiu et al. [52] developed a machine learning framework based on density-functional theory to target 500 highly selective materials from 70,000 COFs, with a membrane selectivity as high as 248, and pointed out that the PLD and LCD are the core parameters for CH_4_/H_2_ screening (Figure 4a). In addition, Cao et al. [53] quantified the differential effects of porosity and the PLD on selectivity and permeability for the separation of isobutylene (i-C_4_H_8_) and 1,3-butadiene (C_4_H_6_) using an RF model. They screened out adsorption-dominated efficient separation membranes from 601 COFs, providing a theoretical basis for hydrocarbon purification. Beyond hydrocarbon separation, COF-based membranes also exhibit unique advantages in the efficient removal of acid gases from natural gas. Xin et al. [41] developed a new method for the rapid screening of high-performance COF-based membranes for the separation of acid gases (H_2_S and CO_2_) from natural gas through combining interpretable machine learning and molecular simulation (Figure 4b). Through molecular simulation and machine learning modeling, porosity was found to be a key factor in determining membrane performance. Using machine learning modeling, the rapid screening of potential high-performance materials from nearly 70,000 hypothetical COFs accelerated the development of COF-based membranes for sour gas separation applications.

#### 3.1.4. Rare Gas Processing

**Figure 5:**
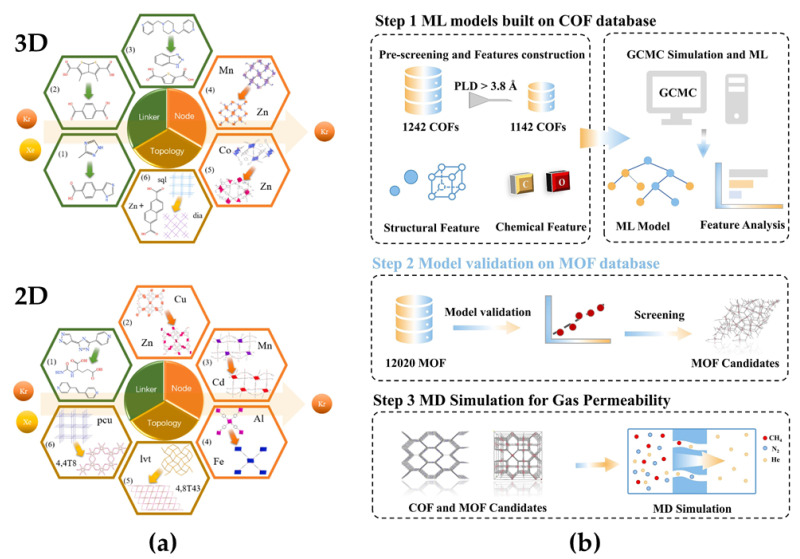
(**a**) Design strategies of 3D and 2D MOF membranes for boosting Kr/Xe separation. Reprinted with copyright permission from Ref. [54] Copyright 2019 Elsevier Ltd.; (**b**) a workflow for developing universal machine learning models to screen high-performance COFs/MOFs membrane materials for helium separation. Reprinted with copyright permission from Ref. [42] Copyright 2024 Elsevier Ltd.

Rare gases are widely used in medicine, lighting, electrical engineering, and aerospace. Xenon (Xe), one of the high-demand rare gases, is currently purified mainly through costly and energy-intensive distillation [36]. To reduce Xe purification costs, researchers have applied various organic framework membranes to Xe separation. Huang et al. [54] utilized HTCS and five ML algorithms (RF, DT, SVM, k-nearest neighbors, and XGBoost) to analyze the structure–performance relationships of 6013 MOF membranes for Kr/Xe separation. They identified key descriptors like PLD and proposed three design strategies to enhance separation performance (Figure 5a). The study combined HTCS, ML, and molecular fingerprints to offer new insights for developing high-performance membranes for Kr/Xe separation. Beyond noble gas separation (e.g., Kr/Xe), helium recovery from natural gas or industrial byproducts has emerged as a critical challenge due to its high economic value and scarcity. Lang et al. [42] developed a universal ML model to predict helium separation performance in COF membranes (Figure 5b). By integrating grand canonical Monte Carlo (GCMC) simulations with machine learning, the model extracted structural (e.g., pore volume, porosity) and chemical descriptors (e.g., isosteric heat of adsorption Qst) of COFs to predict helium adsorption capacity and selectivity. MD simulations further validated the diffusion selectivity of helium in candidate materials, with 3D-Sp-COF-1 exhibiting a threefold enhancement compared to conventional benchmarks. This work highlights the synergy between multiscale simulations and ML in decoding rare gas separation mechanisms, offering a template for accelerating the discovery of energy-efficient purification technologies.

### 3.2. OFMs for Liquid Separations

Compared with the gas separation field, the application of machine learning in liquid separation membranes is still relatively limited, and this gap stems from the complexity of the liquid separation process and the high cost of data acquisition. Liquid separation not only involves complex mechanisms such as multiphase flow dynamics, interfacial interactions, and pollutant adsorption, but also needs to cope with environmental variables such as solution ionic strength and pressure fluctuations, which puts higher requirements on the pore size distribution, chemical stability, and surface functionalization of membrane materials. In addition, the dispersion and lack of standardization of experimental data further limit the generalization ability of the model. Nevertheless, with the combination of machine learning algorithms and multi-scale simulation techniques, this field is gradually showing breakthrough potential.

**Figure 6:**
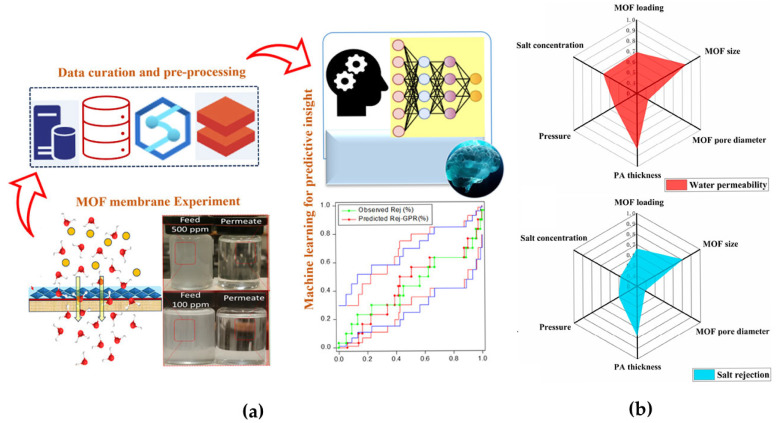
(**a**) An in situ fabrication of a hierarchical PDA-stabilized UiO-66-NH_2_ membrane with machine learning-optimized oil/water separation performance. Reprinted with copyright permission from Ref. [55] Copyright 2024 American Chemical Society; (**b**) the relative importance of input variables for membrane performance: water permeability and salt rejection. Reprinted with copyright permission from Ref. [56] Copyright 2024 Elsevier Ltd.

Usman et al. [55] designed a polydopamine-modified UiO-66-NH_2_ (PDA-s-UiO-66-NH_2_) membrane to address the challenge of water-in-oil emulsion separation and used a Gaussian process regression (GPR) model to predict the permeate flux and oil retention (Figure 6a). It was found that GPR significantly outperformed the SVM and decision tree models in terms of prediction performance by virtue of its nonlinear fitting ability and uncertainty estimation advantages. This work not only validates the applicability of machine learning in complex liquid separations but also reveals the importance of data-driven design for membrane surface engineering. In another study on water treatment membranes, Zhang et al. [56] significantly improved the performance prediction accuracy of MOF-based composite membranes by optimizing a back-propagation (BP) neural network via a genetic algorithm (GA). The study determined the network structure through cross-validation and hyper-parameter tuning, and used a GA to optimize the initial weights and thresholds, which enabled the model to outperform the traditional BP model and algorithms such as RF and SVM in the prediction of water permeability (R^2^ = 0.98) and salt retention (R^2^ = 0.99). Grey relation analysis further indicated that the MOF size and the thickness of the polyamide layer were the dominant factors that affected performance (Figure 6b). This finding provides a quantitative basis for balancing the compatibility of MOF fillers with polymer matrices and promotes the rational design of reverse osmosis membranes. The above studies highlight the unique value of machine learning in resolving the multifactorial coupling of liquid separation. The advantages of the GPR model in nonlinear problems and the efficiency of the GA-BP model in parameter optimization reflect the diversity of algorithmic adaptation scenarios.

Membrane fouling, a pervasive and detrimental obstacle in liquid separations, refers to the deposition, adsorption, or growth of contaminants on the membrane surface or within its pores, leading to flux decline, increased energy consumption, and shortened membrane lifespan. Its primary forms include organic fouling, biofouling, and scaling. Machine learning is emerging as a powerful tool for predicting, monitoring, and mitigating fouling [57]. For instance, Garakani et al. explore how to use physical information neural network (PINN) models to enhance our understanding and predictive capabilities regarding membrane fouling phenomena [28]. Researchers combined physical laws with neural networks to develop a PINN model capable of dynamically allocating weights to different fouling mechanisms. This model not only quantifies the relative importance of each fouling mechanism in flux decay but also accurately predicts flux decay even with limited data, demonstrating higher predictive accuracy and adaptability than traditional machine learning models. This research provides new tools and methods for optimizing membrane filtration systems and improving the efficiency of membrane technology in practical applications. This integration of fouling prediction with cleaning strategy optimization through ML opens new avenues for developing smarter and more fouling-resistant liquid separation systems based on OFMs.

### 3.3. Industrial Translation Pathways

Machine learning is becoming the core engine that is accelerating OFM commercialization by bridging lab-to-factory gaps. Studies have shown that by optimizing CO_2_/CH_4_ separation in membranes with an ANN model, and through case simulations of separating CO_2_/CH_4_ mixtures in raw natural gas, landfill gas, and shale gas, IRMOF-1 membranes achieve higher recovery rates than commercial polymer PTMSP membranes with smaller membrane areas, reaching target purity [25]. This confirms the feasibility of the proposed integrated framework, offering guidance for applying MOF-based materials in industrial gas separation and insights for material design and process optimization. In water treatment, the integration of ML with fluorescence spectroscopy and mechanistic models has provided an innovative approach for predicting membrane flux decline [29]. This enables the effective handling of complex fouling phenomena, improves the efficiency of membrane filtration processes, reduces maintenance costs, and offers new development directions for water treatment technologies. Real-time monitoring and the dynamic adjustment of filtration parameters can optimize membrane filtration, prolong the membrane lifespan, and cut overall operational costs. In seawater desalination, the GA-BP neural network model offers crucial guidance for designing high-performance OFM-based reverse osmosis membranes and serves as a reference for developing other membrane materials [56]. This provides a more economical and feasible solution for freshwater supply in coastal water-scarce regions and helps mitigate global water scarcity.

In summary, ML has played a pivotal role in the industrialization of OFM membranes, accelerating the transition from lab research to industrial application. Through data-driven custom production, ML enables the optimized design and manufacturing of OFM membranes to meet specific industrial demands across different fields. This customization not only enhances the market competitiveness of OFM membranes but also delivers more precise and efficient solutions for related industries. Additionally, ML speeds up the R&D and innovation cycle, allowing OFM membranes to quickly adapt to market changes and continuously introduce high-performance products to maintain technological leadership.

## 4. Machine Learning-Enabled Multiscale Optimization of Membrane Systems

Optimizing membrane separation efficiency requires not only refining the structural properties of membranes but also decoding microscopic mechanisms such as molecular transport dynamics and material–environment interactions. Machine learning indirectly elevates membrane performance by uncovering molecular diffusion patterns, quantifying adsorption–desorption energy barriers, or predicting material responses under extreme operational conditions. For instance, dynamic pore fluctuations in flexible frameworks [54], interfacial modification effects of ionic liquids [50], and molecular diffusion pathways within channels [58]—complex phenomena once intractable—can now be systematically resolved through the integration of ML and multiscale simulations [59]. Notably, the application of machine learning in the field of solid-state electrolytes (SSEs) has also provided useful insights into the study of organic framework membranes. In SSE research, machine learning has successfully established a quantitative relationship between structural features and ionic conductivity properties by analyzing the crystal structure and ion transport paths of the materials, and this data-driven screening method significantly shortens the material development cycle and reduces the experimental cost [60]. Similarly, in the study of organic framework membranes, we can learn from this efficient screening strategy and use machine learning to quickly identify organic framework membrane materials with potentially high performance and optimize their separation performance. This cross-disciplinary borrowing and application fully demonstrates the potential and significant value of machine learning for a wide range of applications in materials science, marking a paradigm shift from phenomenal description to mechanism-driven optimization, and bridging the gap between basic science and industrial implementation.

**Figure 7:**
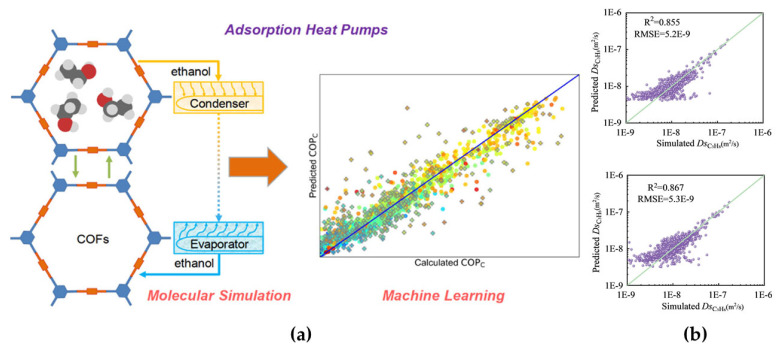
(**a**) The computational and machine learning approach for evaluating COFs in ethanol-based adsorption heat pumps. Reprinted with copyright permission from Ref. [61] Copyright 2020 American Chemical Society; (**b**) regression models for predicting the diffusion coefficients C_3_H_8_ (top) and C_3_H_6_ (bottom). Reprinted with copyright permission from Ref. [58] Copyright 2025 Elsevier Ltd.

The transformative potential of ML in advancing membrane systems lies not in the direct manipulation of membrane structures but in its unparalleled capacity to optimize the multiscale phenomena governing separation processes. By decoding the intricate relationships between molecular building blocks, dynamic transport behaviors, and macroscopic performance, ML acts as an invisible architect, reshaping membrane science from serendipitous discovery to mechanism-driven design. This paradigm shift transcends traditional boundaries, enabling membranes to evolve from static sieves into adaptive, self-optimizing systems tailored for industrial complexity. At the heart of this revolution is ML’s ability to accelerate material discovery—a critical first step toward high-performance membranes. For instance, a RF model trained on a database containing more than 12,000 COFs and MOFs by Li et al. [61] identified the top adsorbents for adsorption-driven heat pumps (AHPs) with 92% accuracy, which reduced the computational cost by two orders of magnitude. These ML-prioritized materials, such as MOFs with pore-limiting diameters of >1 nm, are subsequently integrated into MMMs, indirectly enhancing CO_2_/CH_4_ separation efficiency by optimizing filler–polymer interfaces (Figure 7a). This material-centric strategy not only bypasses trial-and-error synthesis but also establishes a foundation for designing membranes with built-in molecular recognition capabilities.

Beyond material screening, ML unravels the dynamic interplay between molecules and frameworks—a realm where traditional methods falter. Pan et al. [58] analyzed the effect of framework flexibility on the diffusion coefficients of C_3_H_8_ and C_3_H_6_ using MD simulations. The results show that it is difficult to intuitively judge the change of diffusion coefficients when the trends of the PLD and LCD are inconsistent, so the SISSO algorithm, which has strong interpretability, was used to build a model to accurately predict the effect of framework flexibility on diffusion. A prediction model for C_3_H_8_ and C_3_H_6_ diffusion coefficients was also developed. The results emphasize that the prediction of molecular diffusion coefficients in COFs requires a combination of factors such as pore structure, material density, and cell volume (Figure 7b).

From accelerating adsorbent discovery to engineering adaptive pores and resilient frameworks, ML operates as the silent catalyst of membrane innovation. It redefines membranes as intelligent ecosystems where selectivity, permeability, and longevity coexist through data-driven harmony. This convergence of computational intelligence and materials science not only addresses current challenges in carbon capture and water purification but also paves the way for next-generation membranes capable of evolving with the ever-shifting demands of sustainable industries.

## 5. Outlook

The integration of ML with OFMs heralds a transformative era in membrane science, yet challenges and opportunities coexist. While ML has revolutionized gas separation by decoding structure–performance relationships and accelerating material discovery, its application in liquid-phase separations remains nascent due to the inherent complexity of multiphase interactions, dynamic environmental variables, and scarce standardized datasets. Beyond the extensively studied MOFs and COFs, emerging frameworks such as CMPs, POCs, and PAFs offer untapped potential. These materials, characterized by their unique topological flexibility, modular assembly, and chemical diversity, present novel avenues for tailored separation processes. However, the ML-driven exploration of CMPs, POCs, and PAFs remains limited, hindered by insufficient experimental datasets and unclear structure–property correlations. Future advancements hinge on expanding ML applications to these systems and leveraging their distinct advantages, such as CMPs’ tunable π-conjugated networks for photoresponsive separations or POCs’ molecular recognition capabilities for selective ion transport. Enhanced collaboration between computational and experimental communities will be pivotal for establishing unified databases encompassing synthesis conditions, dynamic operational parameters, and multiscale performance metrics. Such efforts will not only improve model generalizability but will also enable the development of adaptive ML frameworks capable of addressing real-world complexities, such as fluctuating pH, pressure gradients, and competitive adsorption in industrial settings.

Interpretability and trust in ML models must be prioritized to translate computational insights into actionable design principles. Tools like SHAP analysis and physics-informed neural networks could unravel the mechanistic origins of membrane behavior, guiding the rational functionalization of pore walls or the stabilization of flexible frameworks under harsh conditions. For instance, ML could optimize the hierarchical porosity of PAFs for high-flux gas separation or decode the host–guest interactions in POCs to enhance chiral separation efficiency. Concurrently, the integration of in situ characterization techniques with ML workflows will unlock dynamic, real-time optimization of membrane systems, transitioning them from static sieves to responsive, self-adapting platforms.

Industrial scalability remains a critical frontier. While ML-driven high-throughput screening has identified promising OFM candidates, their translation into cost-effective, durable membranes demands interdisciplinary collaboration. Innovations in automated synthesis, defect engineering, and hybrid membrane architectures (e.g., mixed-matrix membranes) must align with ML predictions to balance selectivity, permeability, and mechanical robustness. Notably, CMPs and PAFs, with their exceptional thermal stability and processability, could serve as robust fillers or standalone membranes for harsh industrial environments. Furthermore, the convergence of ML with emerging technologies, such as digital twins for process simulation or AI-guided robotic labs for autonomous experimentation, could redefine the pace and precision of OFM development across diverse frameworks.

In addition, the emerging field of PIML holds great potential for bridging the gap between pure data fitting and fundamental physical interpretation in OFM design and simulation. First, it can enhance generalization and robustness. By adhering to underlying physical laws, models reduce the risk of overfitting sparse or noisy experimental data and can make more reliable extrapolations beyond the training data distribution. Second, it can reduce data dependency. Physical laws provide inherent regularization, potentially enabling models to make accurate predictions on smaller datasets—a critical advantage for complex scenarios such as liquid separation or dynamic operating conditions. Third, it can improve interpretability. The model’s solutions naturally follow physical principles, making prediction results more trustworthy and mechanistic insights easier to obtain. PIML represents a key frontier direction. Its development is crucial for achieving truly predictive, multi-scale OFM system digital twins, which will accelerate the rational design of next-generation separation membranes in real dynamic environments.

Ultimately, the fusion of ML and OFMs promises to transcend traditional trade-offs, enabling membranes tailored for sustainability-driven applications like carbon neutrality, water security, and clean energy. By expanding ML’s scope beyond MOFs and COFs to embrace CMPs, POCs, and PAFs, researchers can unlock a broader chemical space for next-generation separation technologies. Through transforming serendipity into strategy, this synergy will not only address global separation challenges but will also catalyze a paradigm shift toward intelligent, eco-efficient industrial systems.
